# Ferroptosis: unveiling a transformative perspective in the landscape of autoimmune diseases

**DOI:** 10.3389/fimmu.2026.1726566

**Published:** 2026-03-30

**Authors:** Shengkuan Chen, Yongruo Cheng, Wangen Li, Yunjuan Zhao

**Affiliations:** 1 Department of Emergency, The Second Affiliated Hospital of Guangzhou Medical University, Guangzhou, China; 2 The Second Affiliated Hospital of Guangzhou Medical University, Guangzhou, China; 3 Department of Endocrinology, The Second Affiliated Hospital of Guangzhou Medical University, Guangzhou, China

**Keywords:** autoimmune diseases, ferroptosis, immune cells, oxidative stress, therapeutic strategies

## Abstract

Ferroptosis, fundamentally defined as an iron-catalyzed and lipid peroxidation-driven cell death process, constitutes an underlying disease mechanism in autoimmune pathogenesis. Recent studies demonstrate that ferroptosis not only shapes innate and adaptive immune responses-including T and B lymphocytes, macrophages, neutrophils, and dendritic cells-but also remodels inflammatory microenvironments to either preserve tissue integrity or exacerbate damage. Pharmacological modulation of ferroptosis, via iron chelators, GPX4 activation, and lipid peroxidation blockade, hold promise as adjuncts to current immunomodulatory approaches. Collectively, ferroptosis represents both a unifying pathogenic framework and a translational target for precision interventions in autoimmunity.

## 1 Introduction

Autoimmune disorders represent a prominent class of pathologies defined by a breakdown in self-tolerance, where a dysregulated immune system attacks host tissues, thereby initiating localized or systemic inflammatory damages (1, 2). The pathophysiology of these conditions typically involves a triad of pathological events: structural compromise of cells and tissues, consequent functional deficits, and sustained inflammatory activity. Collectively, autoimmune disorders afflict an estimated 3-5% of individuals worldwide and are now understood to be more common than historically recognized. Classic autoimmune disorders encompass include Systemic lupus erythematosus (SLE), Rheumatoid arthritis (RA), Vitiligo, Toxic Diffuse Goiter (Graves’ Disease), Type 1 Diabetes Mellitus (T1DM), Multiple Sclerosis (MS), Inflammatory Bowel Disease (IBD), Hashimoto’s Thyroiditis (HT), and Psoriasis (3). Other conditions such as Addison’s disease (AD), Sjogren’s syndrome (SS), Polymyositis (PM), progressive systemic sclerosis, and certain types of glomerulonephritis are also linked to autoimmunity (3–5). The symptoms of these diseases vary due to different causes and the specific organs and tissues involved. Innovations in medical imaging have enhanced diagnostic precision and longitudinal monitoring in autoimmune disorders. However, the integrated immunopathogenic cascades of autoimmune diseases remain partially defined knowledge frontiers. Emerging evidence underscores ferroptosis as a pivotal pathogenic mechanism in several autoimmune conditions, including RA, SLE, T1DM, and others mentioned above (6). The discovery of iron inhibitors (such as AS) (7) has delivered diagnostically-informative therapeutic candidates for autoimmune disease (8).

## 2 Mechanism of ferroptosis

Ferroptosis, a term coined by Dixon et al. in 2012 (8), is defined as a regulated form of cell death precipitated by iron-catalyzed accumulation of lipid peroxides. Mitochondria are central hubs for iron death. Recent studies have reinforced the involvement of mitochondria in ferroptosis, following the discovery that hyperoxidized peroxiredoxin 3 (PRDX3) serves as a robust biomarker for this cell death process in both model organisms and cell cultures (9). The buildup of lipid peroxides within mitochondria induces the hyperoxidation of PRDX3. Following this modification, PRDX3 relocates to the plasma membrane, subsequently impairing cystine import and thereby driving ferroptotic cell death (9). Compared to type 4 immune responses, type 2 immune responses play an accelerated role in autoimmune diseases, and similarly, mitochondria serve as the pivotal orchestrator. During Type 2 immunity, the upregulation of airway epithelial 15-lipoxygenase-1 (15LO1) partners with phosphatidylethanolamine binding protein 1 (PEBP1), forming a complex that shifts substrate specificity toward membrane phospholipids to catalyze pro-ferroptotic 15-HpETE-PE synthesis, a metabolite normally reduced by glutathione peroxidase 4 (GPX4) to its inert alcohol form (10).

Systemic iron homeostasis depends critically on two primary sources: the acquisition of dietary iron through intestinal absorption and the retrieval of this metal from senescent erythrocytes. Hepcidin, a liver-produced hormone, which functions as a pivotal catalytic hub within regulatory cascades. Elevations in systemic iron levels trigger a corresponding upregulation in the synthesis of the regulatory hormone hepcidin (11). Subsequently, hepcidin binding induces the endocytosis and proteolytic degradation of ferroportin, thereby limiting the efflux of iron from intestinal enterocytes into systemic circulation (12). Under conditions of iron deficiency, signaled by low transferrin saturation or ferritin levels, hepcidin expression is repressed. This disinhibition markedly boosts intestinal iron uptake by augmenting its transfer from enterocytes into circulation. Concurrently, multicopper ferroxidases-primarily intestinal hephaestin and plasma ceruloplasmin-catalyze the essential oxidation of Fe^2+^ to Fe^3+^, a prerequisite for systemic iron transport (13). The resulting ferric iron (Fe^3+^) is subsequently chelated by transferrin, a key plasma glycoprotein, which enables its safe and targeted delivery to tissues via the systemic circulation (14). Experimental models involving the tissue-specific genetic ablation of GPX4-such as in renal tissue or immune cells like T and B lymphocytes-have been shown to trigger ferroptosis, underscoring the indispensable function of this pathway in developmental processes (13).

As central regulators, mitochondria coordinate ferroptotic progression by modulating iron homeostasis, amplifying reactive oxygen species (ROS), and undergoing functional compromise. Simultaneously, a consortium of additional organelles-including the endoplasmic reticulum, peroxisomes, Golgi apparatus, and lysosomes-advances this cell death pathway through their involvement in lipid remodeling, antioxidant balance, and the autophagic turnover of ferritin (15). Recent advancements in covalent inhibitor design have successfully targeted these critical cysteine residues, opening new therapeutic avenues for modulating ferroptosis. The YEATS domain protein GAS41, a recognized oncogene and histone reader, partners with transcription factor NRF2 to co-activate ferroptosis regulators such as SLC7A11, promoting cell survival, while its own recruitment to the SLC7A11 promoter is mediated by H3K27-acetylation binding independently of the NRF2 pathway (16). GAS41 functions as a molecular scaffold that stabilizes the association between NRF2 and acetylated H3K27, effectively tethering this transcription factor to specific genomic loci to enable context-dependent gene activation. Thus, the ability of GAS41 to modulate ferroptosis directly enhances its tumorigenic potential, as evidenced in relevant *in vivo* models (17).

GPX4, in conjunction with its active-site selenocysteine (Sec46), contains seven other cysteines (Cys2, Cys10, Cys37, Cys66, Cys75, Cys107, and Cys148), all potentially reactive with electrophiles. The canonical GPX4 inhibitors RSL3 and ML162, which belong to the chloroacetamide class of compounds, were originally designed to covalently engage the catalytic selenocysteine residue (Sec46) through their reactive alkyl chloride groups (18). Structure-based optimization guided by computational modeling of RSL3 and ML162 binding to GPX4 enabled the rational design of the more potent covalent inhibitor, C18 (15). In cellular models utilizing MDA-MB-231 cells, the C18 inhibitor demonstrates superior potency, engaging the Sec46 residue with a half-maximal inhibitory concentration (IC50) of 0.12 μM (19). This binding affinity is markedly greater than that observed for RSL3 (IC50 = 8.42 μM) and ML162 (IC50 = 4.84 μM) (20). The pro-ferroptotic activity of C18 is evidenced by its efficacy in triggering this form of cell death not only in triple-negative breast cancer (TNBC) cell cultures but also in murine xenograft models (16). C18 also demonstrates improved pharmacokinetic properties coupled with a substantially enhanced safety margin, as reflected by its median lethal dose (LD_50_) of 338.3 mg/kg (21). Thereby, C18 demonstrates significantly enhanced efficacy and metabolic stability in inducing ferroptosis compared with RSL3 or ML162 (22). Hepatic deficiency of the fat mass and obesity-associated (FTO) gene augments m^6A RNA methylation on transcripts encoding ACSL4 and TFRC, thereby stabilizing these positive regulators of ferroptotic cell death (23). Lysophosphatidylcholine acyltransferase 1 (LPCAT1) suppresses ferroptosis through membrane lipid remodeling. By elevating saturated phospholipid content via the Lands cycle, LPCAT1 reduces the abundance of peroxidation-prone polyunsaturated fatty acids in membranes, thus mitigating lipid peroxidation and associated cellular damage (24). Dual targeting of LPCAT1 and ferroptosis induction synergistically triggers ferroptosis and suppresses tumor growth (24).

Ferroptosis fundamentally diverges from classical apoptosis, exhibiting unique morphological, biochemical, and genetic characteristics (25, 26). The ferroptosis agonist Erastin triggers this cell death pathway by disrupting mitochondrial integrity, particularly through peroxidation of the electron transport chain that generates excessive reactive oxygen species in an ion-dependent manner (27). Pharmacological inhibition of system xc^-^ by compounds like Erastin and sulfasalazine (SSZ) blocks cellular cystine import, initiating a cascade of intracellular cysteine depletion, glutathione (GSH) exhaustion, GPX4 dysfunction, and consequent ferroptotic cell death (28). This transporter is a heterodimeric complex composed of the SLC7A11 (xCT) and SLC3A2 subunits (29). Beyond its established connections with upregulated lysosomal activity, glutaminolysis, and tricarboxylic acid cycle function, ferroptosis can transition to an apoptotic phenotype when driven by intense endoplasmic reticulum stress and a metabolic shift favoring phosphatidylcholine over phosphatidylethanolamine (30).

SOX13 exerts transcriptional control over SCAF1, facilitating the reorganization of electron transport chain complexes into superassemblies that enhance mitochondrial energetics and cellular resilience against ferroptosis (31). The consequent augmentation of respiratory chain supercomplex formation enhances mitochondrial oxidative phosphorylation and metabolic energy production, thereby fortifying cellular resistance to both chemotherapeutic agents and immunotherapy (32). This ferroptosis-resistant state can be reversed by the antiviral agent zanamivir, which directly binds SOX13 and triggers its proteasomal degradation via TRIM25-mediated ubiquitination (31). Limiting cystine availability promotes the build-up of lipid peroxides within mitochondria, whereas suppression of mitochondrial electron transport effectively blocks phospholipid peroxidation and subsequent ferroptotic death (33). The oxidative destruction of plasma membrane phospholipids constitutes the defining terminal phase in the ferroptotic process (34). Nuclear Protein 1 (NUPR1) is a pivotal regulator of processes like transcription and stress responses, centrally governing redox-ferroptosis interplay; its cisplatin-induced expression depends on RNF113A, and inhibition by ZZW-115 impairs mitochondrial TFAM, diminishing antioxidant defense to sensitize pancreatic cancer cells to ferroptosis through metabolic targeting (35). Research by Liu and colleagues revealed that NUPR1 directly upregulates LCN2 expression, thereby modulating cellular iron homeostasis, suppressing ferroptotic death, and consequently accelerating pancreatic tumorigenesis (36). Recent studies establish that the deubiquitinase USP7 stabilizes Stearoyl-CoA Desaturase (SCD) by removing its ubiquitin chains, thereby connecting USP7 loss-of-function to accelerated SCD breakdown and subsequent activation of ferroptotic cell death (37).

This conclusion was reached via an integrated multi-omics approach, incorporating metabolomic and transcriptomic profiling, alongside validation in a lung epithelial-specific Cpt1a-deficient murine model and corroborating clinical data. Within the lung tumor microenvironment, CPT1A—a master regulator of fatty acid β-oxidation—cooperates with macrophage-derived L-carnitine to orchestrate a dual outcome: conferring resistance to ferroptosis while simultaneously impairing the antitumor function of CD8^+^ T lymphocytes (38). Mechanistically, a positive feedback loop is established between CPT1A and c-Myc, wherein CPT1A stabilizes the c-Myc oncoprotein by limiting its ubiquitin-mediated proteasomal turnover, and in turn, c-Myc enhances CPT1A gene transcription (38). This self-reinforcing CPT1A/c-Myc signaling circuit establishes robust ferroptosis protection in cancer stem cells through dual mechanisms: bolstering the cellular antioxidant defense via NRF2-mediated GPX4 upregulation and limiting peroxidation-susceptible phospholipid pools through ACSL4 suppression (38). Loss of WIPI4 function promotes ATG2A accumulation at ER-mitochondria membrane contact sites, thereby stimulating phosphatidylserine transport into mitochondrial compartments (39). Accelerated phosphatidylserine transport into mitochondria serves as a critical metabolic switch that upregulates intramitochondrial synthesis of phosphatidylethanolamine-a phospholipid highly susceptible to peroxidation-thereby establishing a permissive environment for ferroptosis initiation. The ATG2A-mediated phosphatidylserine import mechanism exhibits minimal overlap with classical ferroptosis stimuli (39).

**Figure 1:**
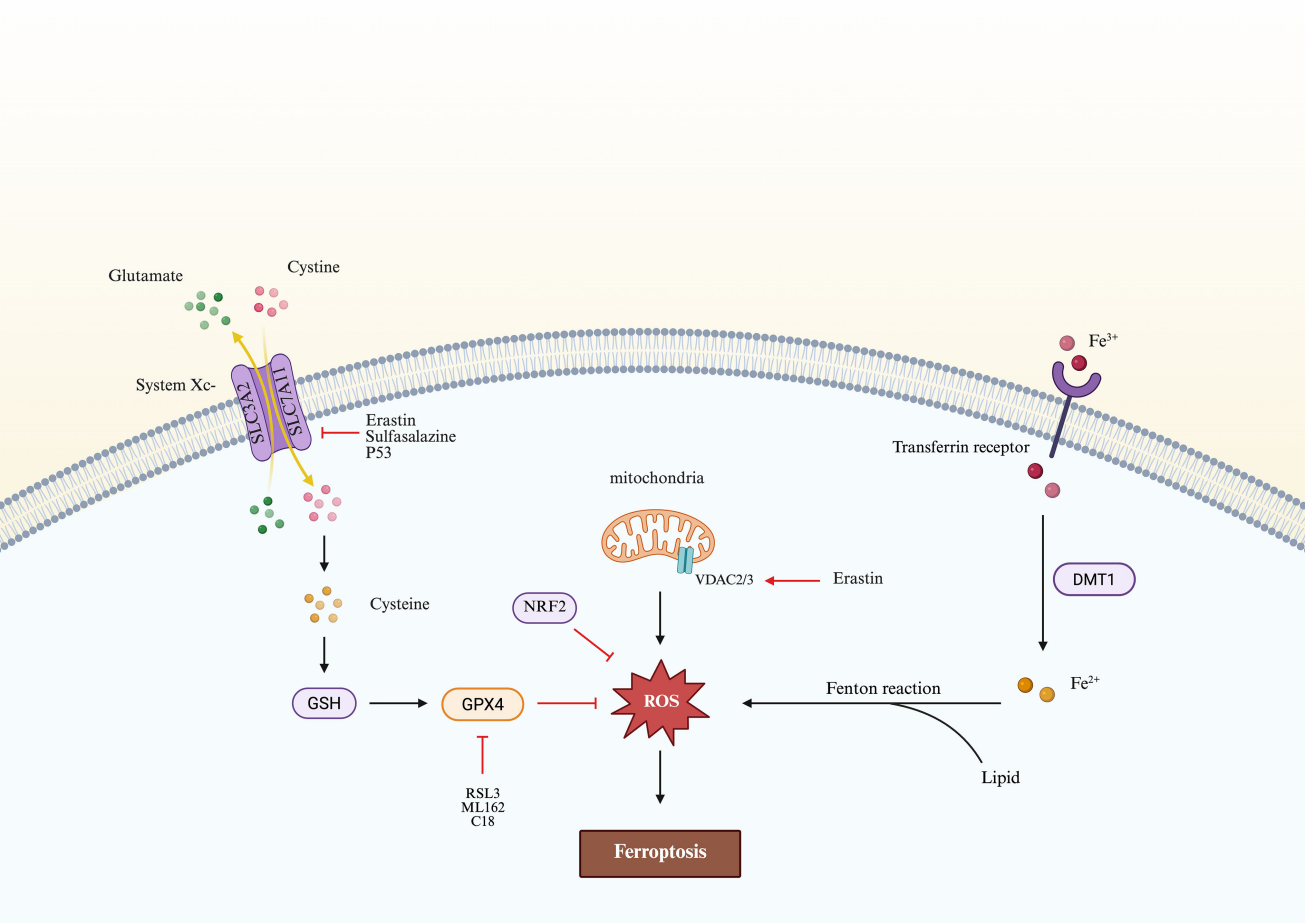
Mechanism of ferroptosis: diagram illustrating the essential molecular events driving ferroptotic cell death. System Xc^-^ mediates the exchange of extracellular cystine and intracellular glutamate, providing cysteine for glutathione (GSH) synthesis. By employing reduced glutathione (GSH) as an essential cofactor, glutathione peroxidase 4 (GPX4) catalyzes the detoxification of lipid hydroperoxides into corresponding alcohols, thus effectively suppressing membrane lipid peroxidation and subsequent ferroptotic cell death. Inhibitors such as erastin, sulfasalazine, and p53 suppress System Xc^-^ activity, while compounds like RSL3, ML162, and C18 directly inhibit GPX4, leading to accumulation of ROS. Iron uptake via transferrin receptor and divalent metal transporter 1 (DMT1) increases the labile iron pool, which participates in the Fenton reaction to generate ROS. Mitochondrial dysfunction, including voltage-dependent anion channel (VDAC) modulation, further amplifies ROS production. Collectively, excessive ROS-mediated lipid peroxidation triggers ferroptosis.

**Figure 2:**
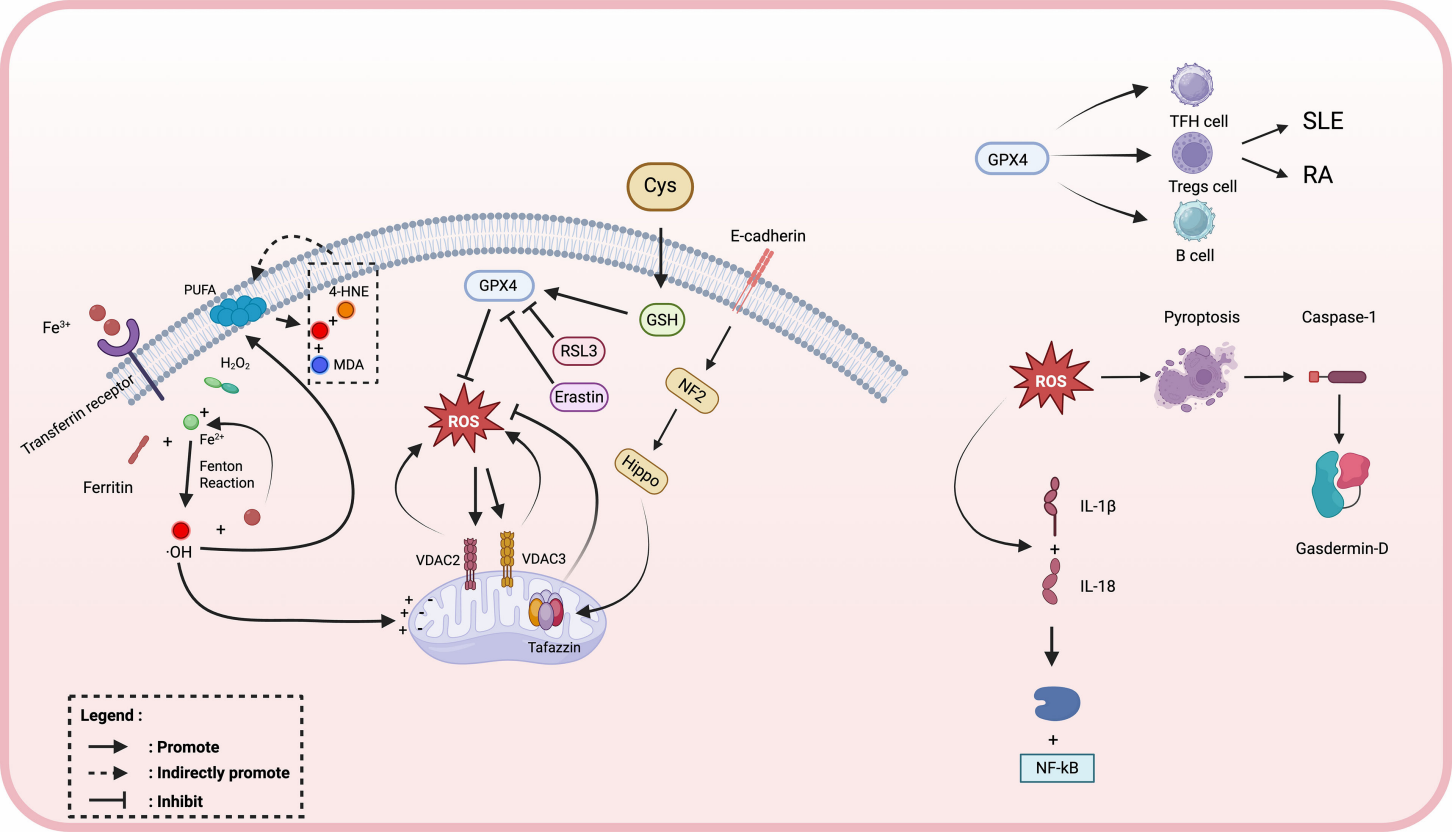
Key molecular pathways of ferroptosis: crosstalk between ferroptosis and immune regulation in autoimmune diseases. Intracellular iron accumulation via the transferrin receptor promotes Fenton reaction–mediated hydroxyl radical (•OH) generation, driving lipid peroxidation of polyunsaturated fatty acids (PUFAs) into toxic aldehydes such as 4-hydroxynonenal (4HNE) and malondialdehyde (MDA). This process is amplified by mitochondrial dysfunction and voltage-dependent anion channels (VDAC2/3) activity, while inhibited glutathione peroxidase 4 (GPX4) activity—through agents such as RSL3 or erastin—reduces lipid peroxide detoxification. Cysteine depletion and GSH deficiency further enhance ROS accumulation. Elevated ROS levels can activate pyroptosis via caspase-1–mediated gasdermin D cleavage, leading to IL-1β and IL-18 release and downstream NF-κB activation. Reactive oxygen species (ROS) derived from ferroptotic and pyroptotic pathways dysregulate the function of pivotal adaptive immune populations-encompassing T follicular helper (Tfh) cells, regulatory T (Treg) cells, and B lymphocytes-thereby driving the pathogenesis of RA and SLE.

Structurally characterized by up to four BCL-2 homology (BH) domains that govern protein interactions, the BCL-2 protein family is divided into three functionally opposing categories: multi-domain pro-survival members (e.g., BCL-2, BCL-XL, MCL-1), which inhibit apoptosis by sequestering BH3-only initiators (such as BIM, PUMA) and multi-domain effectors (BAX/BAK); through this regulated network, the family controls mitochondrial membrane integrity and BAX/BAK pore formation, thereby executing intrinsic apoptosis (40). The detailed mechanism of ferroptosis can be seen in **Figure 1** and **Figure 2**. Specific molecular pathways and their roles in ferroptosis are summarized in **Table 1**.

**Table 1:** Key molecular pathways involved in ferroptosis.

Pathway/molecule	Mechanism and role in ferroptosis
GPX4	GPX4 is essential in ferroptosis which catalyzes the reduction of lipid peroxides to non-toxic lipid alcohols, thereby preventing lipid peroxidation and cell death (41).
SLC7A11 (Cystine/Glutamate Antiporter)	This antiporter system imports cystine into the cell in exchange for glutamate, which is critical for the synthesis of glutathione (GSH). GSH serves as a cofactor for GPX4 (42).
Erastin and RSL3	Erastin inhibits SLC7A11, diminishing cystine uptake and GSH synthesis, while RSL3 directly inhibits GPX4, both of which lead to elevated lipid peroxidation and ferroptosis.
VDAC2/3 (Voltage-Dependent Anion Channels)	VDAC2/3, as targets of ferroptosis inducers like Erastin, modulate mitochondrial ion and metabolite flux, thereby influencing reactive oxygen species (ROS) generation and cellular redox balance (8, 27, 43–46). VDAC2/3-mediated mitochondrial dysfunction amplifies lipid peroxidation and promotes the opening of permeability transition pores, contributing to the loss of mitochondrial membrane integrity and subsequent plasma membrane damage during ferroptosis; however, suppression of mitochondrial electron transport or loss of mitochondrial mass effectively blocks ferroptosis, whereas cell death triggered by GPX4 inhibition remains unaffected, suggesting context-dependent roles of mitochondrial pathways in different ferroptotic stimuli (47).
NF2 and Hippo signaling pathway	Cell-cell contact induces E-cadherin, which suppresses ferroptosis by activating NF2 and the Hippo pathway. Deletion of downstream tafazzin confers ferroptosis resistance. NF2 inactivation increases ferroptosis sensitivity, highlighting a translational target (48, 49). The disruption of cellular integrity during ferroptosis results from nanoscale pores within the plasma membrane, whereas the intercellular transmission of this death signal requires erastin treatment but does not occur following GPX4 blockade (34).

## 3 The significance of ferroptosis in autoimmune diseases

Originally identified as a mechanism to induce cell death in tumors, ferroptosis also plays a pivotal role in other conditions such as sepsis (50, 51). In addition to pervasive lipid peroxidation, emerging research suggests that genomic instability significantly promotes ferroptosis, where impaired macrophage migration inhibitory factor (MIF) or homologous recombination repair factors such as BRCA1 heighten susceptibility by disrupting DNA damage response mechanisms (52). The pivotal involvement of ROS in ferroptosis underscores its importance in the onset and progression of organ damage (15, 43, 53). Emerging evidence links ferroptosis to the apoptosis and autophagy of immune cells, suggesting its relevance in autoimmune diseases (54). Functioning independently of its canonical unfolded protein response role, the endoplasmic reticulum-resident protein IRE1α governs ferroptotic susceptibility through an evolutionarily conserved mechanism wherein its endoribonuclease activity degrades transcripts encoding glutathione biosynthesis factors GCLC and SLC7A11, rendering IRE1α-deficient cells resistant while overexpressing cells hyper-sensitive to this death pathway (55).

The functional states of immune and stromal cells are directly governed by their metabolic configurations, wherein microenvironmental cues reprogram core metabolism to dictate functional responses, while activation-induced functional shifts conversely demand adaptive metabolic remodeling (56). Dysregulated ferroptosis mediates pathogenic progression in autoimmune disorders by causing excessive immune cells death or dysfunction. Targeting specific molecular pathways of ferroptosis constitutes a promising therapeutic strategy.

In human hepatocellular carcinoma cell lines, both genetic or pharmacological disruption of DHCR7 and external administration of its metabolic substrate 7-dehydrocholesterol (7-DHC) effectively prevented the onset of ferroptosis (57). Delving into the mechanisms of ferroptosis in immune cells has paved the way for new diagnostic markers and treatment strategies, driving significant advancements in the management of autoimmune diseases (58). Despite its promise as a combinatory approach with conventional cancer treatments, ferroptosis induction carries risks of adverse effects such as myelosuppression, hepatorenal toxicity, and secondary tumor development (59).

## 4 Ferroptosis and immune cells

### 4.1 T cells and B cells

Ferroptosis, a type of iron-dependent cell death, involves distinct mechanisms in T cells and B cells (60–63).

Ferroptosis in CD8^+^ T cells is mediated by IFN-γ which is produced by activated CD8^+^ T cells and NK cells (64). The combined action of IFN-γ and arachidonic acid (AA) triggers peroxidative damage within malignant cells, ultimately resulting in tumor cell death (65). This mechanism is critical as it operates alongside traditional cytotoxic pathways such as granzyme-perforin and Fas-FasL. CD4^+^ T cells include TFH cells (Follicular Helper T cells) and Regulatory T Cells. TFH cells, are crucial in regulating germinal centers and promoting immune responses post-vaccination and infection. Ferroptosis in TFH cells is influenced by the GPX4 axis (66). Ferroptosis and pyroptosis function as pivotal regulators in TFH cell homeostasis, with the AKT-GSK3β axis involved in inhibiting ferroptosis, thereby prolonging the survival of CD4^+^ T cells. Additionally, mitophagy, induced by ferroptosis, regulates CD8^+^ T cell apoptosis and can be modulated by IL-15, impacting diseases such as Parkinson’s (65, 67–72). Regulatory T Cells (Tregs): GPX4-maintained redox balance is essential for regulatory T cell (Treg) function, as its deficiency disrupts immune homeostasis through lipid peroxidation-mediated impairment, potentially driving pathogenesis in autoimmune diseases like SLE and RA (73).

B cells are particularly sensitive to lipid peroxidation, which is a hallmark of ferroptosis. GPX4 is essential in regulating ferroptosis within B cells (22). Furthermore, IL-6 produced by renal neutrophils can inhibit ferroptosis in B cells (64). Inhibition of IL-6 significantly promotes ferroptosis, suggesting potential therapeutic strategies for conditions like lupus nephritis. Marginal Zone B Cells and B1 Cells exhibit higher sensitivity to lipid peroxidation and ferroptosis, highlighting their role in autoimmunity. The sensitivity of these cells to ferroptosis could be leveraged to modulate autoimmune responses (74). Long-lived plasma cells rely on GPX4 for survival (75). Ferroptosis inhibition in long-lived plasma cells might provide a strategy to prolong antibody responses in vaccination or autoimmune diseases where excessive autoantibody production is a concern (45, 50, 74–78).

Research has demonstrated that IFN-γ and AA are necessary for inducing lipid peroxidation and subsequent ferroptosis in tumor cells targeted by CD8^+^ T cells. This pathway adds a new dimension to the cytotoxic mechanisms of CD8^+^ T cells, complementing existing pathways. Among B lymphocyte populations, B1 and marginal zone subsets exhibit heightened susceptibility to peroxidative damage and ferroptotic cell death (79–82). Additionally, lymphocyte lines are prone to GPX4-regulated ferroptosis, providing new avenues for treating diseases linked to B cell proliferation and differentiation (83). Possessing stem-like properties, tumor-repopulating cells (TRCs) develop therapeutic resistance to both chemotherapy and irradiation through mechanisms that reduce their susceptibility to ferroptotic cell death (81). Employing quantitative mass spectrometry and lipidomics, researchers identified that the mitochondrial kinase PCK2 mediates the phosphorylation and functional activation of ACSL4, thereby orchestrating membrane phospholipid reorganization linked to ferroptosis (84). By suppressing PCK2, tumor-repopulating cells (TRCs) undergo structural and metabolic adaptations that establish a cellular state refractory to ferroptosis (85). The ferroptosis-associated heterogeneity in host cells for the first time, which suggests that ferroptosis may become a new clinical target for resisting bacterium infection (86).

### 4.2 Macrophages and neutrophils

In macrophages and neutrophils, ferroptosis involves in distinct molecular pathways. Macrophages play dual roles in ferroptosis, influencing inflammatory responses and tissue homeostasis. Lipid ROS activates MAPK and NF-κB signaling pathways in M1 macrophages, promoting inflammation. Suppression of ferroptosis in M1 macrophages may prolong their pro-inflammatory effects, which is relevant to autoimmune diseases such as multiple sclerosis (MS) and inflammatory bowel disease (IBD) (87, 88). M2 macrophages are generally resistant to ferroptosis due to higher GPX4 expression. However, targeting ferroptosis in M2 macrophages could modulate their function in chronic inflammatory diseases (89).

Extracellular vesicles (EVs)-encompassing exosomes, microvesicles, retrovirus-like particles, and apoptotic bodies-serve as key intercellular mediators that inhibit ferroptosis through activation of the Nrf2 pathway and subsequent upregulation of the antioxidant gene SLC7A11 (90). Inhibiting macrophage-capping protein suppresses UFM1-mediated pirin modification, which downregulates GPX4 transcription and promotes HMGB1 cytosolic translocation, ultimately triggering ferroptosis and rapid release of this damage-associated molecular pattern (91). The modified HMGB1 release dynamics promote macrophage polarization toward an M1-like phenotype, rendering MCP inhibition a dual-therapeutic strategy that concurrently induces ferroptosis and activates antitumor immunity (92).

Lipid peroxidation constitute a pivotal mechanism in ferroptosis, varying across different organs, tissues, and cells (93). Ferroptosis contributes to NET formation, exacerbating inflammation in diseases such as SLE (87). As an essential subunit of the NADPH oxidase complex, NCF1 is required for neutrophil ROS generation, and its genetic variations are linked to increased susceptibility to autoimmune disorders including rheumatoid arthritis and lupus (91).

Lipid reactive oxygen species engage MAPK and NF-κB signaling cascades to centrally modulate inflammatory processes, as established by current research (94). Macrophages are serves as indispensable mediators in this process, acting as pro-inflammatory agents. Emerging evidence underscores the pivotal involvement of ferroptosis in the pathogenesis of multiple autoimmune diseases. In MS, the application of ferroptosis inhibitors has shown promise in reducing neurodegeneration (90). As a member of the leukocyte immunoglobulin-like receptor family, the immunoreceptor LILRB1 contains immunoreceptor tyrosine-based inhibition motifs and interacts with the MHC class I subunit β2-microglobulin on tumor cells (92). This LILRB1-β2-microglobulin engagement elicits broad immunosuppression across multiple leukocyte populations; for instance, genetic ablation of LILRB1 on macrophages enhances their phagocytic clearance of tumor cells, thereby potentiating antitumor immunity (95).

### 4.3 Other immune cells involved in ferroptosis

Dendritic Cells (DCs) exert a pivotal function in antigen presentation and immune modulation. Ferroptosis regulates DCs function by altering lipid metabolism and antigen processing. The GPX4 pathway influences DCs survival, and ferroptotic stress may compromise T-cell activation, affecting immune tolerance (91, 96).

Natural Killer (NK) Cells mediate cytotoxicity against infected and tumor cells. Ferroptosis in NK cells can be triggered by excessive lipid peroxidation, impairing their function (97). Enhancing ferroptosis resistance in NK cells may improve their efficacy in cancer immunotherapy (97–99).

Innate Lymphoid Cells (ILCs) particularly ILC2s, participate in allergic responses and tissue repair. Ferroptosis regulation in ILCs is an emerging area, with implications for diseases such as asthma and fibrosis (100–102).

**Figure 3:**
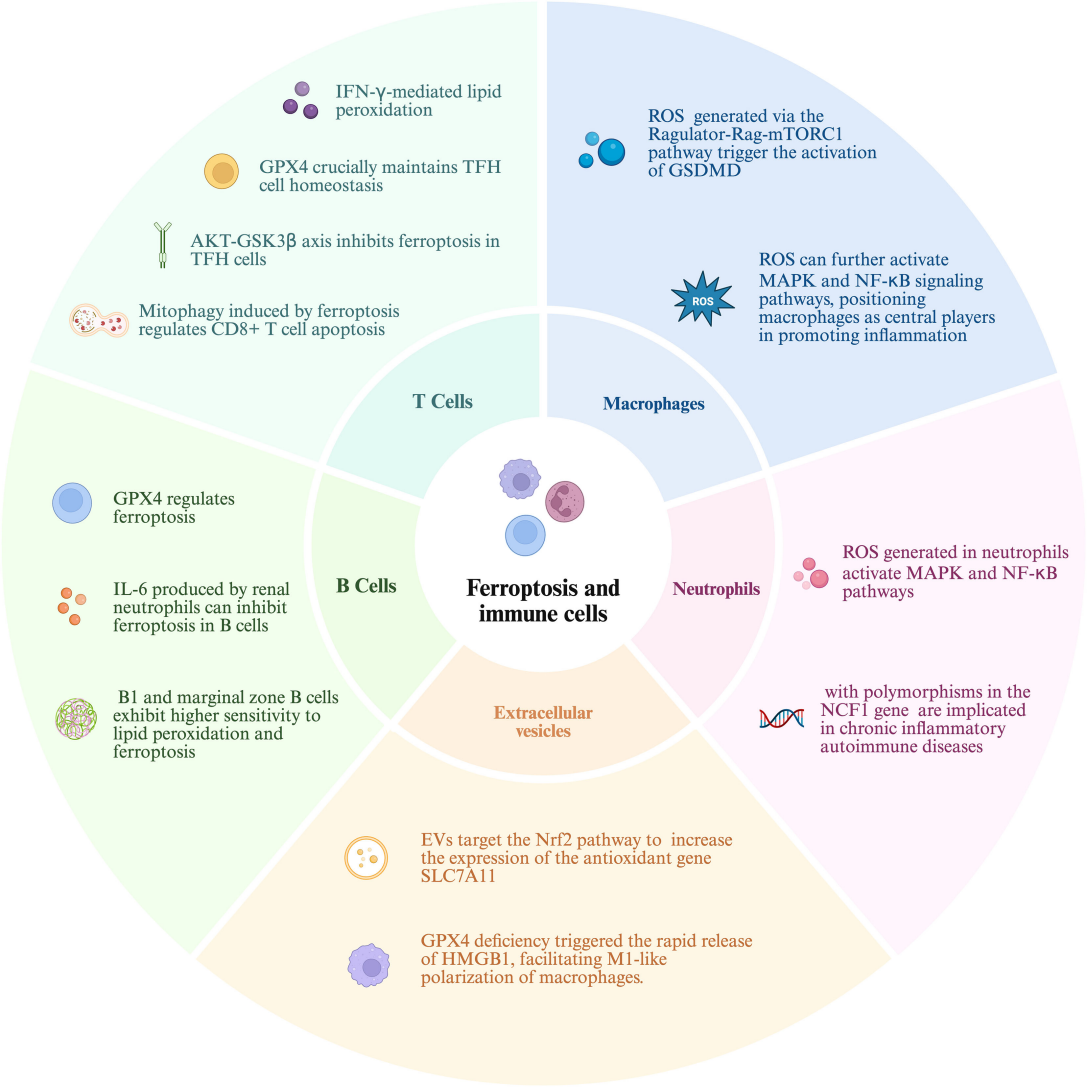
The relationship between immune cells and ferroptosis mechanisms: ferroptosis-mediated regulation of different immune cell subsets in autoimmune diseases. In T cells, IFN-γ–induced lipid peroxidation and AKT–GSK3β signaling modulate ferroptosis, with GPX4 maintaining T follicular helper (TFH) cell homeostasis and mitophagy regulating CD8^+^ T cell apoptosis. Within B lymphocytes, ferroptosis is governed by GPX4 activity, suppressed by neutrophil-derived IL-6, and exhibits heightened susceptibility in B1 and marginal zone subsets due to increased lipid peroxidation. In macrophages, ROS generated via the Ragulator–Rag–mTORC1 pathway activates gasdermin D (GSDMD)–mediated pyroptosis, while ROS also enhances MAPK and NF-κB signaling, driving pro-inflammatory activation. In neutrophils, ROS triggers MAPK and NF-κB pathways, and NCF1 gene polymorphisms are linked to chronic inflammatory autoimmune diseases. Extracellular vesicles (EVs) can modulate ferroptosis by activating the Nrf2 antioxidant pathway through SLC7A11 expression, whereas GPX4 deficiency promotes HMGB1 release and M1-like polarization of macrophages.

Ferroptosis represents a novel and intricate form of programmed cell death that interacts with various immune cells, influencing their function and survival. Understanding the distinct mechanisms of ferroptosis in T cells, B cells, macrophages, and neutrophils not only expands our knowledge of immune regulation but also opens up potential therapeutic options for autoimmune diseases. Our review aims to explore these complex interactions, shedding light on potential interventions that could revolutionize the treatment of autoimmune conditions (64, 103–109). The relationship between immune cells and ferroptosis mechanisms is shown in **Table 2** and **Figure 3**.

**Table 2:** The relationship between immune cells and ferroptosis mechanisms.

Immune cell type	Ferroptosis mechanism	Key molecules/signaling pathways
T Cells	Ferroptosis induced by IFN-γ-mediated lipid peroxidation (66)	IFN-γ, GPX4, SLC7A11
B Cells	Ferroptosis regulated by GPX4 (74)	GPX4, Erastin, RSL3
Macrophages	Ferroptosis through ROS generation and lipid peroxidation (87)	ROS, MAPK, NF-κB
Neutrophils	Ferroptosis through lipid peroxidation and inflammatory responses (93)	ROS, GSDMD, MAPK

## 5 Ferroptosis and autoimmune diseases

### 5.1 Systemic lupus erythematosus

Ferroptosis exhibits substantial pathogenic contributions to SLE via iron-dependent lipid peroxidation and oxidative stress. This process involves the formation of malondialdehyde (MDA) and 4-hydroxynonenal (4-HNE) from the peroxidation of polyunsaturated fatty acids (PUFAs) (110)in cell membranes. Endoplasmic reticulum membranes are enriched with polyunsaturated fatty acid-containing phospholipids (PUFA-PLs), highlighting the pleiotropic nature of lipids which function as cell death mediators, signaling molecules, and metabolic substrates—a functional diversity underpinned by the lipidome’s immense structural variation across tissues, cell types, and organelles (111). Disruption of lipid homeostasis underlies the pathogenesis of numerous human diseases, highlighting its fundamental role in preserving cellular health and systemic physiology (112). Reactive metabolites from lipid peroxidation disrupt essential cellular structures, initiating cell death and DAMP release—a process further amplified by antioxidant depletion and exemplified in SLE, where neutrophils undergo ferroptosis and NETosis, activating TLR-dependent ROS generation that propagates inflammatory tissue injury (76, 87, 113–115)​. The polyunsaturated nature of PUFAs enables critical signaling and membrane fluidity functions while conferring peroxidation susceptibility, rendering membranes rich in PUFA-phospholipids-synthesized by ACSL4 and LPCAT3-vulnerable to ferroptosis through iron-catalyzed Fenton reaction-mediated oxidation (116).

Ferroptosis inhibitors like liproxstatin-1 and deferoxamine can attenuate neutrophil death and reduce oxidative damage (117). Targeting GPX4 and NRF2 pathways, which regulate antioxidant defenses, may provide effective strategies to mitigate ferroptosis and its pathological effects in SLE. Research demonstrates that these inhibitors can significantly reduce lipid peroxidation and protect against cellular damage in lupus nephritis models, offering a promising adjunctive therapy to traditional immunosuppressants (110)​. Renal tubular cells exhibit upregulated Snord3a levels during lupus nephritis pathogenesis (112). Snord3a transcriptionally activates STING signaling, and its targeted inhibition with antisense oligonucleotides demonstrates therapeutic efficacy in murine acute kidney injury models (118). Beyond GPX4’s protection against lipid peroxide-mediated death in conditions like lupus nephritis, the transcription factor NRF2 bolsters cellular redox defenses, while in heart failure, renal proximal tubules sustain high ATP demand through a finely-regulated iron cycle—involving transferrin endocytosis, DMT1 transport, mitochondrial use, ferritin storage, and ferroportin export—to conserve iron and prevent its urinary loss (119).

Combining these inhibitors with traditional immunosuppressants like corticosteroids or hydroxychloroquine may improve treatment efficacy and patient outcomes by addressing both immune dysregulation and oxidative damage in SLE (1, 2, 120)​.

### 5.2 Rheumatoid arthritis

In RA, TNF-α serves as a key mediator of ferroptosis through iron-dependent lipid peroxidation. This mechanism potentiates reactive oxygen species generation through dual pathways: NADPH oxidase (NOX) activation and mitochondrial electron transport chain impairment (121–123). This leads to lipid peroxidation of PUFAs in synovial fibroblasts, causing cell death. Bioinformatics analysis has further identified key ferroptosis-related genes (e.g., GPX4, ACSL4, SLC7A11) and their correlation with immune cell infiltration characteristics in RA, providing molecular evidence for the involvement of ferroptosis in synovial inflammation and immune dysregulation (124). The death of these fibroblasts releases pro-inflammatory cytokines like IL-1β and IL-6, which recruit and activate macrophages and T cells. These immune cells perpetuate chronic inflammatory responses and mediate progressive joint destruction. Mitochondrial impairment serves as a key driver of ferroptosis by elevating reactive oxygen species and promoting the iron-mediated Fenton process, wherein ferrous ions facilitate the transformation of hydrogen peroxide into highly reactive hydroxyl radicals, thereby exacerbating oxidative damage and triggering ferroptotic cell death (121, 125, 126)​​. Dysregulated iron homeostasis amplifies ferroptotic susceptibility through Fenton chemistry-driven lipid peroxidation and polyamine synthesis via the ODC1-MYC axis, a metabolic and oxidative cascade prominently observed in the inflamed synovium of rheumatoid arthritis (72).

Inducing ferroptosis in specific cell populations using agents like Imidazole ketone erastin (IKE) can suppress synovial fibroblast proliferation and inflammation (11). This approach might disrupt the inflammatory cycle in RA (64). Combining ferroptosis inducers with existing RA therapies could enhance treatment efficacy and minimize side effects (104). This combination strategy aligns with emerging interdisciplinary perspectives, such as the theory of “Strengthening Body Resistance (Fú Zhèng)” in traditional Chinese medicine, which emphasizes modulating co-inhibitory receptor-regulated T cell immunity to restore immune homeostasis in RA (127). For example, targeting the GPX4 pathway in synovial fibroblasts can prevent lipid peroxidation and subsequent cell death, reducing inflammation and joint damage. Additionally, leveraging NRF2 activation can bolster cellular antioxidant defenses, further mitigating the pathological effects of ferroptosis. Studies have shown that integrating these approaches with standard RA treatments like methotrexate or biologics may offer a comprehensive strategy to manage RA by addressing both immune dysregulation and oxidative stress​ (78, 128, 129).

### 5.3 Vitiligo

Vitiligo involves heightened oxidative stress and iron accumulation, which makes melanocytes highly susceptible to ferroptosis. Elevated ROS, produced by mitochondrial ETC and NADPH oxidase, initiate lipid peroxidation of PUFAs in melanocyte membranes. Reactive aldehydes including malondialdehyde (MDA) and 4-hydroxynonenal (4-HNE), formed during lipid peroxidation, exert cytotoxic effects that ultimately lead to cell death. Cytokines such as IFN-γ, CXCL9, and CXCL10 recruit cytotoxic T cells to skin lesions, exacerbating oxidative stress and inducing ferroptosis in melanocytes (130). Depletion of key antioxidants including glutathione and catalase exacerbates melanocyte susceptibility to both oxidative injury and ferroptotic cell death (131). This process results in the characteristic depigmented patches of vitiligo​ (103, 104, 130–133)​​.

Antioxidants like selenium and vitamin E can attenuate oxidative stress and protect melanocytes from ferroptosis. Functioning as a selenium shuttle, PRDX2 delivers selenide to SEPHS2 to enhance GPX4 and other selenoprotein expression, thereby antagonizing ferroptosis and creating a permissive environment for cancer development (134). Targeted suppression of ferroptosis using compounds like ferrostatin-1 and liproxstatin-1 protects melanocytes from demise, thereby ameliorating vitiligo pathology (133). Targeting mitochondrial ROS production through mitochondrial-targeted antioxidants like MitoQ, and enhancing antioxidant defenses through NRF2 activators, are promising strategies for vitiligo treatment (135). Integrating these approaches with conventional treatments such as phototherapy or topical corticosteroids may offer a comprehensive therapeutic strategy to manage vitiligo by protecting melanocytes from oxidative stress and ferroptosis (136). Additionally, boosting the expression of GPX4, an enzyme that reduces lipid hydroperoxides, could further protect melanocytes from ferroptosis and oxidative damage, leading to improved repigmentation and stabilization of the condition (15, 135–138)​.

### 5.4 Toxic diffuse goiter (Graves’ disease)

In Graves’ Disease (GD) and its ocular manifestation, Graves’ Orbitopathy (GO), ferroptosis affects cell metabolism and iron balance. Autoantibodies stimulate thyroid cells to produce excess thyroid hormones, leading to hyperthyroidism (50, 54, 64, 133, 139). This hypermetabolic state results in enhanced glycolysis and increased ROS production, exacerbating oxidative stress. Despite the high levels of iron present, GO fibroblasts resist ferroptosis due to robust antioxidant defenses, particularly the elevated activity of GPX4 and NRF2 (nuclear factor erythroid 2–related factor 2). A recent study demonstrated that primary GO orbital fibroblasts exhibited stronger resistance to ferroptosis induced by cystine deprivation or erastin compared to control fibroblasts, and selenium—which enhances GPX4 activity—protected control fibroblasts from ferroptotic cell death (140). These antioxidant pathways help neutralize ROS and prevent lipid peroxidation, allowing fibroblasts to survive. The survival and proliferation of these fibroblasts contribute to tissue remodeling and inflammation characteristic of GO​​ (141, 142).

Modulating ferroptosis by targeting metabolic pathways offers promising therapeutic strategies for GD and GO (143, 144). For instance, using IGF1R inhibitors to inhibit glycolysis can reduce ROS production and promote ferroptosis in GO fibroblasts, thereby alleviating symptoms. Additionally, targeting the NRF2 pathway to diminish its activity can help overcome the resistance of GO fibroblasts to ferroptosis. Enhancing ferroptosis in these resistant cells could potentially reduce tissue remodeling and inflammation. Combining these strategies with traditional treatments for hyperthyroidism, such as antithyroid drugs or radioiodine therapy, may provide a more comprehensive approach to managing GD and GO​ (140, 145).

### 5.5 Type 1 diabetes mellitus

In T1DM, ferroptosis significantly contributes to pancreatic β-cell death and the autoimmune response. Iron accumulation and lipid peroxidation in β-cells, driven by mitochondrial dysfunction and inflammatory cytokines like IL-1β and IFN-γ produced by T cells and macrophages, lead to β-cell dysfunction and apoptosis (105, 106, 109, 146). Elevated ROS levels promote lipid peroxidation and ferroptosis, exacerbating the autoimmune attack and reducing insulin production. The mitochondrial electron transport chain (ETC) and NADPH oxidase (NOX) are key sources of ROS in β-cells, and their dysfunction further promotes oxidative stress. Additionally, inflammatory cytokines amplify this stress by activating signaling pathways, such as the NF-κB pathway, that enhance ROS production and lipid peroxidation. Iron metabolism critically intersects with oxidative stress pathways, as iron overload potentiates reactive oxygen species production via Fenton chemistry, which transforms H_2_O_2_ into highly destructive hydroxyl radicals (•OH)​ (77, 147, 148).

Within the pancreatic islet microenvironment, pro-inflammatory macrophages and disrupted lipid homeostasis characterized by oxidized lipid deposition collectively drive the pathogenesis of both type 1 and type 2 diabetes (110). The constitutive expression of CXCL16 likely represents a metabolic reprogramming in islet macrophages that enables adaptation to the surrounding oxidative stress (112). The chemokine CXCL16 serves as a primary mechanism for oxidized LDL clearance by pancreatic macrophages, while additional pathways also contribute to this scavenging function (113). Beyond CXCL16, additional mechanisms such as low-level CD36 expression, macropinocytotic uptake, and efferocytotic processes may facilitate the clearance of oxidized LDL (114). Such adaptive responses could clarify the link between inherent NOD mouse vulnerabilities and β cell failure mediated by redox imbalance (149).

Ferroptosis inhibitors such as the MD2 inhibitor L6H21 and Irisin can protect β-cells by reducing oxidative stress and preventing cell death. L6H21 works by inhibiting the TLR4/MD2 complex, which is involved in inflammatory signaling and ROS production (115). While this suppression diminishes NF-κB activation and subsequent pro-inflammatory mediator generation, irisin conversely activates the SIRT1-p53-SLC7A11/GPX4 axis to preserve redox balance and inhibit ferroptosis (116). SIRT1 stimulates the deacetylation and functional enhancement of PGC-1α-a pivotal controller of mitochondrial biosynthesis and cellular antioxidant capacity-while p53 concurrently modulates SLC7A11 expression, a core subunit of the cystine/glutamate antiporter essential for glutathione production (117). By converting phospholipid hydroperoxides into harmless alcoholic derivatives, GPX4 effectively inhibits membrane lipid peroxidation and suppresses ferroptotic cell death (118). Developing inhibitors that protect β-cells and other affected tissues could provide new treatment strategies for T1DM (119). Combining these inhibitors with existing diabetes treatments, such as insulin therapy or immunomodulatory drugs, might offer a comprehensive approach to managing T1DM by addressing both autoimmunity and oxidative stress. Additionally, exploring the potential of NRF2 activators to enhance cellular antioxidant capacity could further protect β-cells from ferroptosis. Clinical trials and further research are needed to validate the efficacy and safety of these therapeutic strategies in patients with T1DM (150, 151)​.

### 5.6 Multiple sclerosis

In MS, ferroptosis significantly orchestrates neurodegeneration and demyelination through several interconnected processes. Disrupted iron balance elevates ferrous ion concentrations, driving hydroxyl radical generation through Fenton chemistry that subsequently induces widespread phospholipid peroxidation within neural cells (152). This lipid peroxidation damages cell membranes, leading to cell death (16). Microglia, the resident macrophages of the CNS, and infiltrating T cells exacerbate neuroinflammation by releasing pro-inflammatory cytokines and ROS, further promoting ferroptosis. Key genes involved in these processes include HMOX1 (heme oxygenase 1), which degrades heme to produce iron, carbon monoxide, and biliverdin, thereby contributing to increased intracellular iron levels and oxidative stress (22). Lysophosphatidylcholine acyltransferase 3 (LPCAT3) facilitates membrane phospholipid reorganization essential for lipid peroxide generation, while ribosomal protein L8 (RPL8) modulates iron homeostasis to regulate Fenton reaction substrate availability—both mechanisms converging to promote ferroptotic progression (153). The mitochondria and endoplasmic reticulum (ER) are critical sources of ROS and lipid peroxidation products. Mitochondrial dysfunction leads to the release of ROS, while ER stress contributes to the accumulation of misfolded proteins, both of which amplify neural damage and drive the progression of MS​ (69, 108, 154, 155). If Nervous system damage, phagocytosis of red blood cells results in tremendous iron load in macrophages. Liberated during heme catabolism by HO-1, iron is sequestered within ferritin nanocages in its non-reactive ferric state (Fe^3+^), distinct from the redox-active ferrous form (Fe^2+^) (23). The ferritin nanocage, assembled from 24 FTH and FTL subunits with a capacity for ~4500 iron atoms, releases its stored metal either through selective autophagic degradation (ferritinophagy) or passively upon phagocyte demise during advanced disease, liberating redox-active iron (156).

Ferroptosis inhibitors that enhance antioxidant defenses and block the Fenton reaction have shown efficacy in animal models by improving neurological function and reducing disease severity. N-acetylcysteine (NAC) replenishes intracellular GSH levels and enhances the antioxidant defenses of neural cells. Ferroptosis suppression is achieved through dual mechanisms, GPX4 employs glutathione to detoxify lipid hydroperoxides, while deferoxamine chelates intracellular iron, limiting Fe^2+^ for Fenton chemistry and consequently attenuating reactive oxygen species generation and membrane lipid oxidation (153). Inhibiting G9a, a histone methyltransferase, in neuronal cultures has been found to enhance the expression of antiferroptosis genes, such as those regulated by NRF2. This enhances cellular stress responses and reduces neurodegeneration (76, 157–159). Targeting ferroptosis in MS treatment could provide a new therapeutic avenue by focusing on reducing oxidative stress and protecting neural cells from lipid peroxidation. Combining these strategies with existing MS therapies, such as immunomodulatory drugs, might offer a comprehensive approach to managing MS by addressing both immune dysregulation and oxidative damage​ (125, 160–162). In neurons, STING serves as a targetable central driver that sits at the convergence of neuroinflammation and ionic disruption, underpinning the progression of neurodegeneration (158). Furthermore, the expression levels of other established ferroptosis regulators-ACSL4, SLC7A11, and FSP1-remained unaltered in STING-expressing neurons under both basal conditions and following exposure to glutamate (161). Consequently, STING activation promotes the lysosomal turnover of GPX4 in neurons experiencing concurrent inflammatory stimulation and excitotoxic insult (163).

The IRG1/itaconate axis exerts a neuroprotective role against ferroptosis following intracerebral hemorrhage by covalently modifying cysteine 66 of GPX4, leading to its allosteric activation and enhanced enzymatic function (164).

### 5.7 Inflammatory bowel disease

Inflammatory Bowel Disease (IBD), encompassing Crohn’s disease (CD) and ulcerative colitis (UC), is a chronic inflammatory disorder of the gastrointestinal tract driven by a combination of genetic susceptibility, immune dysregulation, and gut microbiota alterations (165). While the disruption of epithelial integrity and persistent inflammation are well-established contributors to disease pathogenesis, recent findings indicate that ferroptosis serves as a central regulatory node in intestinal epithelial cell (IEC) injury and immune activation (166, 167). Studies have shown that IECs in IBD patients exhibit decreased GPX4 expression and increased susceptibility to lipid peroxidation, leading to ferroptotic cell death, barrier dysfunction, and increased gut permeability (168, 169).

Beyond epithelial damage, ferroptosis contributes to the pro-inflammatory microenvironment in IBD (170). Neutrophils and macrophages infiltrating the inflamed gut release excessive ROS and cytokines such as TNF-α and IL-1β, exacerbating oxidative stress and amplifying ferroptosis-induced tissue injury (171). Moreover, gut dysbiosis alters iron metabolism, with pathogenic bacteria such as Escherichia coli producing siderophores that increase local iron accumulation, further fueling ferroptotic processes (172). This iron-driven oxidative stress sustains chronic inflammation and perpetuates intestinal damage.

Therapeutic interventions targeting ferroptosis have demonstrate therapeutic promise in IBD models. The administration of liproxstatin-1 or ferrostatin-1, which are specific compounds that suppress ferroptosis, shields intestinal epithelial cells from peroxidative damage and aids in the recovery of intestinal barrier integrity (173, 174). Additionally, iron chelators (e.g., deferoxamine, deferiprone) may help mitigate iron-driven oxidative stress, potentially reducing inflammation (175). The activation of NRF2, a key antioxidant regulator, through agents such as sulforaphane or bardoxolone methyl could further enhance cellular defenses against ferroptotic injury, offering a promising avenue for future IBD treatment (173, 176, 177).

### 5.8 Hashimoto’s thyroiditis

Hashimoto’s Thyroiditis (HT) is the most common autoimmune thyroid disorder, characterized by chronic lymphocytic infiltration, progressive thyroid follicular cell destruction, and eventual hypothyroidism (178). Although thyroid hormone production in follicular cells is iron-dependent, iron overload—resulting from hepcidin-mediated ferroportin blockade, macrophage-derived ferritin uptake, and heme catabolism—and resultant oxidative stress can trigger their degeneration through the ferroptosis pathway (179). Studies have indicated that HT thyroid tissues exhibit reduced GPX4 expression, leading to an imbalance in lipid peroxidation and antioxidant defenses, making thyroid cells highly susceptible to ferroptotic death (180, 181).

The immune system further exacerbates ferroptosis in HT. Pro-inflammatory M1 macrophages accumulating in thyroid tissue secrete hepcidin and ferritin, altering iron homeostasis and increasing oxidative stress, which in turn promotes thyroid follicular cell ferroptosis (25). Additionally, Th1 and Th17 immune responses, characterized by elevated levels of IFN-γ and IL-17, contribute to ROS generation and increased lipid peroxidation, perpetuating inflammation and accelerating disease progression (182).

Potential therapeutic strategies focus on reducing oxidative damage and modulating iron metabolism (183, 184). Selenium supplementation, known to enhance GPX4 activity, has demonstrated the ability to reduce oxidative stress and protect thyroid cells from ferroptotic injury (60, 185). Iron chelation therapy, using agents like deferoxamine, may help prevent iron-driven lipid peroxidation, although its effects on thyroid hormone synthesis must be carefully evaluated (186). Targeting ferroptosis pathways through NRF2 activation also holds promise for mitigating oxidative damage and slowing disease progression in HT (187).

### 5.9 Psoriasis

Psoriasis is a chronic autoimmune skin disorder characterized by keratinocyte hyperproliferation, immune cell infiltration, and systemic oxidative stress (188). Recent studies have identified ferroptosis as a contributing factor to keratinocyte dysfunction and inflammation, highlighting the roles of lipid peroxidation, iron dysregulation, and ROS accumulation (189). Conspicuously diminished expression of GPX4 and NRF2 in psoriatic lesions thereby results in compromised antioxidant capacity and a heightened propensity for ferroptosis, consequently aggravating the breakdown of the epidermal barrier (190, 191).

The interplay between ferroptosis and immune activation plays a significant role in psoriasis pathology. Th17-associated cytokines (IL-17, IL-22) stimulate ROS production in keratinocytes, making them more prone to ferroptotic death (192). Additionally, macrophages within psoriatic plaques display dysregulated iron metabolism, contributing to elevated local iron levels and increased oxidative damage. This iron-driven lipid peroxidation exacerbates keratinocyte turnover and immune activation, perpetuating the inflammatory cycle characteristic of psoriasis (193).

The modulation of ferroptotic cell death presents a promising treatment strategy for psoriasis, as evidenced by the efficacy of specific inhibitors such as ferrostatin-1 and liproxstatin-1 in shielding keratinocytes from inflammatory damage in experimental studies (194). Additionally, NRF2 activators such as bardoxolone methyl may enhance cellular antioxidant defenses, reducing ferroptosis-mediated skin damage. Iron chelators (e.g., deferoxamine) are also under investigation to determine their potential role in reducing iron-driven oxidative stress, offering a promising avenue for psoriasis treatment (191, 195–197).

**Figure 4:**
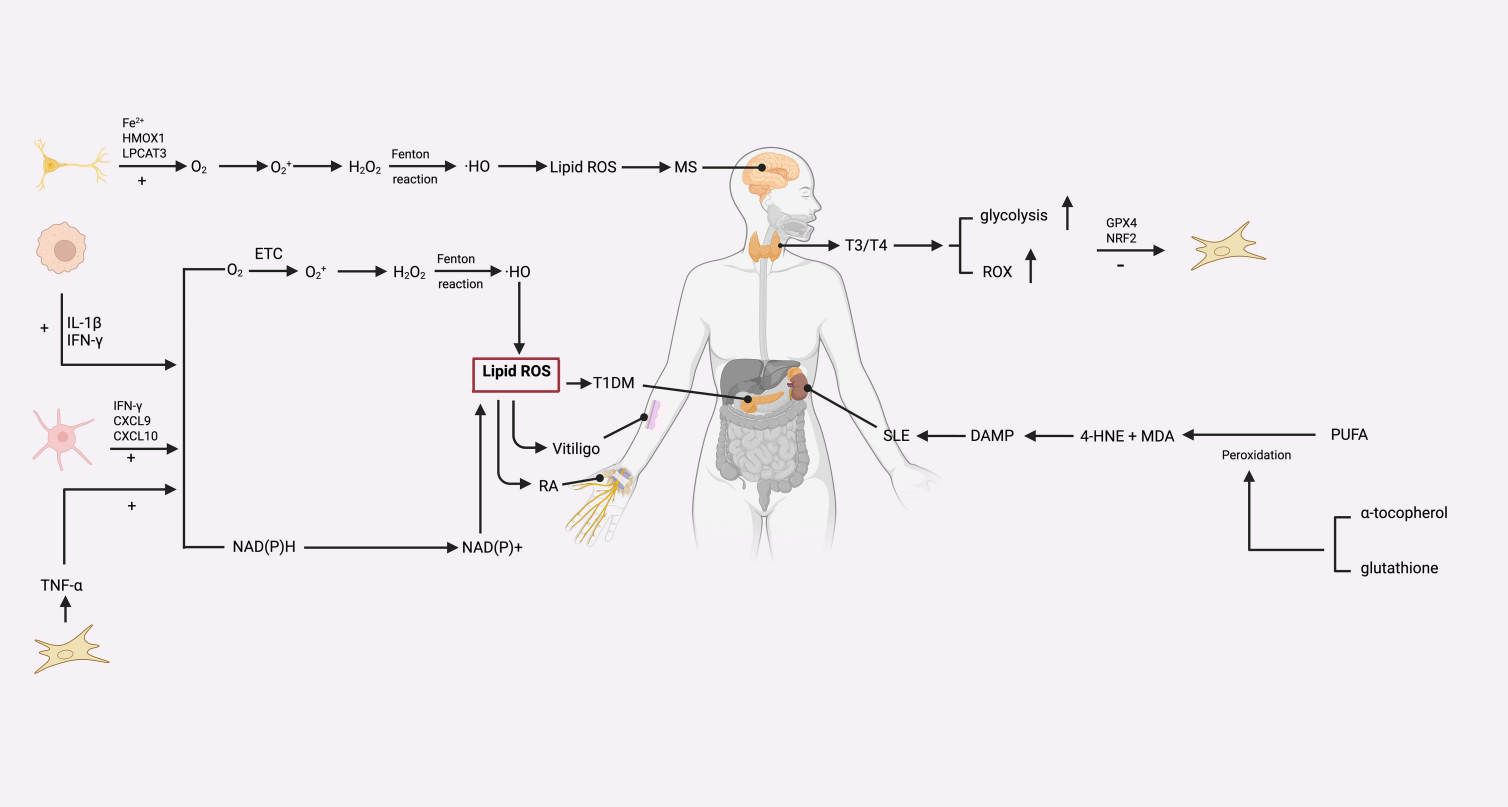
The relationship between autoimmune diseases and ferroptosis mechanisms: ferroptosis-associated lipid peroxidation pathways in systemic autoimmune diseases. In MS, neuronal iron accumulation, HMOX1 activity, and LPCAT3-mediated phospholipid remodeling enhance electron transport chain (ETC)–driven ROS generation, leading to hydroxyl radical (•OH)–mediated lipid ROS accumulation. In T1DM and vitiligo, immune cell–derived IL-1β and IFN-γ amplify mitochondrial ROS production, while chemokines (CXCL9, CXCL10) and TNF-α further potentiate oxidative stress. In RA, increased NAD(P)H oxidase activity promotes lipid peroxidation. In SLE, lipid peroxidation products such as 4-hydroxynonenal (4HNE) and malondialdehyde (MDA) act as damage-associated molecular patterns (DAMPs) to sustain inflammation. In autoimmune thyroid diseases, thyroid hormone (T3/T4)–induced glycolysis elevates ROS, which is counterbalanced by GPX4 and NRF2 antioxidant pathways. Depletion of α-tocopherol, glutathione, and polyunsaturated fatty acids (PUFAs) facilitates ferroptotic injury across multiple autoimmune contexts.

The relationship between Autoimmune Diseases and Ferroptosis Mechanisms is shown in **Table 3** and **Figure 4**.

**Table 3:** The relationship between Autoimmune diseases and Ferroptosis mechanisms.

Autoimmune diseases	Ferroptosis mechanism	Key molecules/signaling pathways
SLE	Ferroptosis through lipid peroxidation and changes in antioxidant levels (110, 119)	MDA (198), α-tocopherol (149), glutathione (75)
RA	Ferroptosis through TNF-α activation in synovial fibroblasts (122)	TNF-α (157), GPX4 (12), Erastin (163)
Vitiligo	Ferroptosis through the death of melanocytes (130)	IFN-γ (103), CXCL9 (104), CXCL10 (132)
GD	Ferroptosis through iron balance dysregulation (141)	TRAb (141), GPX4 (142), SLC7A11 (140)
T1DM	Ferroptosis through the death of pancreatic β-cells (146)	Fe^2+^ (105), GPX4 (106), SLC7A11 (109)
MS	Ferroptosis through iron homeostasis regulation in neural cells (155)	Fe^2+^ (108), GPX4 (160), SLC7A11 (69)
IBD	Ferroptosis through intestinal epithelial cell injury, GPX4 downregulation, and gut dysbiosis-driven iron accumulation (174)	GPX4 (169), ACSL4 (167), Ferroportin (171)
HT	Ferroptosis through hepcidin-mediated ferroportin blockade, macrophage-derived ferritin uptake, and heme catabolism-induced iron overload (179)	GPX4 (172), Hepcidin (35), IFN-γ (182)
Psoriasis	Ferroptosis through keratinocyte GPX4/NRF2 downregulation and Th17 cytokine-driven oxidative stress (189)	GPX4 (190), NRF2 (191), IL-17 (192)

### 5.10 Summary of ferroptosis-targeted therapeutic strategies

Based on the disease-specific discussions above, ferroptosis-targeted therapeutic strategies can be categorized into three main approaches based on their mechanisms of action:

Iron Chelators (e.g., Deferoxamine, Deferiprone) sequester labile iron to limit Fenton chemistry-driven hydroxyl radical generation. They have demonstrated protective effects in SLE (attenuating neutrophil ferroptosis), IBD (preserving intestinal epithelial integrity), and MS (limiting iron availability for neuronal damage).

GPX4 Activators and Antioxidant Enhancers bolster cellular defenses against lipid peroxidation. Selenium supplementation enhances GPX4 activity and shows clinical efficacy in Hashimoto’s Thyroiditis. NRF2 activators (e.g., Bardoxolone Methyl, Sulforaphane) upregulate antioxidant gene programs and have shown promise in IBD and psoriasis. In T1DM, compounds like Irisin activate the SIRT1-p53-SLC7A11/GPX4 axis to protect β-cells.

Lipid Peroxidation Inhibitors (e.g., Ferrostatin-1, Liproxstatin-1, Vitamin E) act as radical-trapping antioxidants, terminating lipid peroxidation chain reactions. They have demonstrated efficacy in SLE (reducing renal damage), IBD (protecting intestinal epithelium), and vitiligo (preserving melanocytes).

Emerging Strategies include metabolic modulation (e.g., IGF1R inhibitors reducing glycolysis in Graves’ Orbitopathy fibroblasts) and selective ferroptosis induction in pathogenic cells (e.g., IKE targeting synovial fibroblasts in RA). Combining these approaches with conventional immunosuppressants offers synergistic potential by addressing both immune dysregulation and oxidative tissue damage.

Clinical translation requires addressing challenges including cell-type specificity, development of disease-specific ferroptosis biomarkers, optimization of combination regimens, and careful safety assessment to avoid unintended effects such as myelosuppression or hepatorenal toxicity.

## 6 Conclusion

This review has highlighted the critical role of ferroptosis in the pathogenesis and progression of various autoimmune diseases, including SLE, RA, Vitiligo, GD, T1DM, MS, IBD, HT, and Psoriasis. Ferroptosis, a unique form of regulated cell death characterized by iron-dependent lipid peroxidation, significantly impacts immune cell function and inflammatory responses, contributing to the autoimmune disease process (87, 88, 141, 199, 200).

Importantly, ferroptosis does not merely add another layer to the cell death landscape but reshapes the conceptual boundaries of autoimmune pathology (201–204). Its bidirectional effects—capable of exacerbating tissue damage when uncontrolled, yet also offering opportunities for immune modulation when precisely targeted—underscore its duality as both a threat and a therapeutic opportunity (205, 206). Advances in pharmacological inhibitors, iron chelators, lipid metabolism modulators, and GPX4-targeted interventions highlight the translational potential of ferroptosis research (207). Integrating these strategies with existing immunotherapies could transform disease management by simultaneously suppressing autoimmunity and restoring tissue integrity (198, 208). Informed by recent studies from 2024-2025, future research should prioritize the development of disease-specific ferroptosis biomarkers, optimization of combination therapy regimens, creation of next-generation modulators with enhanced cell-type specificity and pharmacokinetic properties, and elucidation of ferroptosis crosstalk with other regulated cell death pathways (152).

In conclusion, ferroptosis represents a promising target for the treatment of autoimmune diseases. By advancing our understanding of this unique cell death pathway and developing targeted therapies, we can potentially improve patient outcomes and offer new hope for those suffering from these chronic and often debilitating conditions. Future research in this area holds significant potential to transform the landscape of autoimmune disease treatment.
